# Medical, neurobiological, and psychobehavioral perspectives of mastocytosis: a case report

**DOI:** 10.1186/s13256-021-02757-x

**Published:** 2021-03-30

**Authors:** A. Héron, V. Papillon, D. Dubayle

**Affiliations:** 1 Faculté de Santé, Université de Paris, Physiologie Humaine, 4 avenue de l’Observatoire, 75006 Paris, France; 2 Groupement Hospitalier de Territoire, Unité de Recherche Clinique URC28, Centre Hospitalier Général Victor Jousselin, 44 avenue JF Kennedy, 28100 Dreux, France; 3 Université de Paris, CNRS UMR 8002, Integrative Neuroscience and Cognition Center, 45 rue des Saints Pères, Paris, France

**Keywords:** Mastocytosis, Mind–body connection, Stress, Life events, Case report

## Abstract

**Background:**

Cutaneous mastocytosis is a rare pathology characterized by an abnormal proliferation and degranulation of mast cells, affecting the skin. Here we present the case of a patient suffering from chronic resistant mastocytosis. An original integrative method of evaluation was tested in this patient, to improve therapeutic management. It integrated the interactions between stressful life events and medical history as well as psychobehavioral components and neurobiological factors.

**Case presentation:**

The patient was a 65-year-old Caucasian woman. The cutaneous symptoms of mastocytosis had progressively evolved over the past 36 years, increasingly affecting the patient’s quality of life. At the time of the evaluation, psoralen and ultraviolet A therapy had reduced pruritus, but very unsightly brown-red maculopapules persisted on the chest, back, and arms. We proposed an integrative diagnosis that combined a semistructured interview, a psychometric assessment with the Millon Behavioral Medicine Diagnostic tool, and the collection of medical data. The medical data were compared with the analysis of the significant events in the patient’s life, to determine the threshold of tolerance to stress beyond which the skin symptoms led to profuse thrusts of pruritus. At the same time, the psychobehavioral profile of the patient was determined; this highlighted how social isolation, the denigrated coping style, and problematic compliance could influence the extension of dermatological symptoms. The effects of stressors on the infiltration and degranulation of skin mast cells have been discussed in light of the neurobiological processes currently known. At the end of the evaluation, a new therapeutic strategy was proposed.

**Conclusion:**

This case report reveals the mind–body relationship of a patient suffering from mastocytosis. It highlights the points of vulnerability and the adaptative strategies specific to each patient to be considered in therapeutic management of other resistant chronic diseases.

## Introduction

Harmonious relationships between mind and body are essential to staying healthy. The skin is an organ particularly sensitive to imbalances such as those caused by stressful situations. Mastocytosis is a cutaneous pathology that has not yet been studied with regard to the mind–body relationship. Cutaneous mastocytosis is a disease of the hematopoietic stem cells preferentially occurring in childhood. Cutaneous manifestations most often disappear at puberty. This pathology is due to mast cell proliferation that causes symptoms such as pruritus, characterized by brown maculopapules on the skin [1]. These symptoms are explained by the degranulation of peripheral mast cells [2]. Mast cells can then release proteases such as tryptase and numerous mediators such as heparin, histamine, serotonin, substance P, cytokines, interleukins, lipids, and growth factors​. More than 50 substances can be released from mast cells. Some of them are the cause of the skin manifestations of mastocytosis [3].

Most of mastocytosis is due to a mutation of the gene coding for the c-kit receptor. The c-kit receptor ligand is the stem cell factor (SCF) that induces mast cell proliferation in physiological conditions. In mastocytosis, the c-kit gene mutation leads to overproduction of mast cells, which is the cause of the onset of symptoms [4].

In adults, when mast cells moderately infiltrate other tissues, the pathology is considered as indolent systemic mastocytosis. In this case, “atypical” mutations of the c-kit gene occur and other symptoms usually appear. These symptoms can be chronic (skin lesions, asthma, muscle, joint or abdominal pain, nausea, vomiting, diarrhea) or paroxysmal (flushing, anaphylactic shock, syncope). Neuropsychiatric symptoms have also been described (headaches, attention deficit, auditory or visual memory disorders, depression, sensitivity to stress), suggesting a cerebral effect of mast cells in this pathology [1, 5, 6]. In 10% of cases, systemic mastocytosis becomes aggressive, with a poor prognosis and median survival of 2 to 4 years.

In 90% of cases, the mastocytosis remains cutaneous and treatments are available to reduce symptoms. It is recommended that affected people avoid conditions that trigger or worsen their symptoms when possible. Certain medications such as oral antihistamines and topical steroids are often prescribed [7]. Affected adults may also undergo photochemotherapy, which can help alleviate itching and improve the appearance of the patches [8].

In this study, we present the case of an adult woman with a resistant form of cutaneous mastocytosis. The patient did not develop the systemic form of the pathology, but despite treatments, the cutaneous symptoms were progressively extended for many years, causing significant psychological distress and unaesthetic discomfort. To find an efficient medical care and improve the quality of life of the patient, an original assessment was decided in addition to the clinical diagnosis. This comprehensive and personalized method collects qualitative and quantitative data. It involves psychometric tools and scientific knowledge to evaluate the mind–body relationship.

## Case presentation

The patient was a 65-year-old Caucasian woman, with a history of cutaneous mastocytosis. She was divorced, with two children, and was a retired teacher. The patient did not smoke or drink alcohol. She had no major medical history except the presence of lumbar and femoral osteopenia. A detailed medical examination was performed on admission and revealed very good general condition, weight 54 kg, height 163 cm, blood pressure 120/85 mmHg, pulse 52 beats per minute, body temperature 37.3°C, supple abdomen, without hepatosplenomegaly, and normal cardiovascular, pulmonary, and neurological examinations. She had exhibited symptoms of mastocytosis for 36 years with pruritus and was diagnosed by skin biopsy. Dermatological examination revealed small brown-red maculopapules on the thorax, back, and arms (Fig. 3). A positive Darier’s sign was elicited 5 minutes after skin scratching (the skin becomes swollen, itchy, and red). Additional laboratory analyses were performed. Complete blood count, blood ionogram, and glycemia were normal. No abnormal liver or renal functions were detected. Moderate proteinuria (0,18 g/24 hours) was diagnosed and had to be monitored,. The serum tryptase level was slightly higher than normal (23.3 ng/ml) during crisis. Magnetic resonance imaging showed no evidence of skeletal involvement, and paraclinical examination had concluded the absence of extracutaneous manifestations. The systemic form of the disease had thus been excluded, and the diagnosis of cutaneous mastocytosis had been confirmed. A dermatological treatment was prescribed to the patient: application of a dermocorticoid cream once or twice a day with a maximum of two tubes of creams per month (clobetasol propionate at 0.05%, at the time of skin lesions with prurit). It was supplemented by oral administration of an antiallergic antihistamine drug (levocetirizine dihydrochloride 5 mg, two tablets per day). These local and oral treatments have been ineffective in the long term. Finally, photochemotherapy was also proposed to the patient. Photochemotherapy combines treatment with psoralen and exposure of the skin to long-wave ultraviolet radiation (PUVA). At the time of the assessment, PUVA therapy (30 sessions, 1500 J) had been given each year for 4 years. The sessions had achieved reduction of pruritus with persistence of a moderate Darier’s sign, but very unaesthetic skin lesions had persisted with profuse thrusts. These cutaneous symptoms significantly decreased the quality of life of the patient.

During the medical consultation, the patient was informed of the objectives and procedures of the psychobehavioral assessment of the Millon Behavioral Medicine Diagnostic (MBMD) tool [9–11]. MBMD is a self-report inventory containing 165 true–false items. It provides scales to assess psychobehavioral factors, such as negative health habits, psychiatric indications, coping styles, stress moderators, treatment prognostics, and management guides. The scale aimed to identify attitudes that may affect health of the patient.

At the next visit, after giving consent, the patient told her life history during a structured interview. The Holmes and Rahe Social Readjustment Rating Scale [12] was used to score each stressful life event in “Life Change Units” (LCU). The life events and LCU were collected in an Excel file and classified chronologically. LCU applied to life events for 1 year were summed to assess the annual stress score. In parallel, the medical data of the electronic health records were chronologically integrated in the Excel file to establish a comprehensive dermatological history of the mastocytosis. An automatized graph integrating annual stress scores and cutaneous symptoms was generated.

**Fig. 1:**
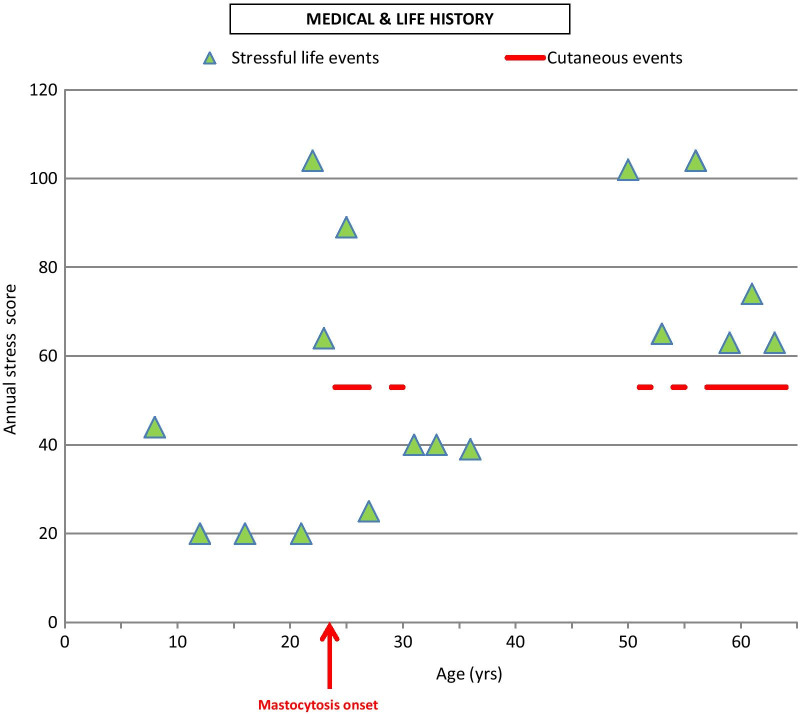
Medical data and stressful life events. Horizontal axis corresponds to the age of the patient (in years). Green triangles represent annual stress scores. Holmes and Rahe Social Readjustment Rating Scale was used to score each stressful life event (in “Life Change Units” LCU). Scores were calculated after the clinical interview of the patient. LCU applied to life events during 1 year were summed to assess the annual stress score. Red lines indicate the periods of cutaneous events recorded in the medical chart. Annual stress scores of life events were low (<50) during childhood. We did not have documentation about the medical status during this period. At the age of 22 years, two major stressful life events occurred: business readjustment (39 LCU) and divorce (65 LCU), totaling a global stress score of 104 LCU. At 23 years old, a second business readjustment (39 LCU) occurred with an abroad change in living conditions (25 LCU) (annual stress score = 39 + 25 = 64 LCU). Mastocytosis crisis (red lines) appeared at the age of 24 years as localized brown cutaneous lesions. It was scored with 53 LCU (according to Holmes and Rahe instructions, pathological events are considered to be stressful and scored with 53 LCU). At the age of 25 years, the patient moved to another country (Africa, 39 LCU) and married (50 LCU) (annual stress score = 89 LCU). The patient contracted subcutaneous filariasis at age 27 years (53 LCU) and developed a Quincke’s edema of unknown origin (53 LCU) at age 30 years. Until her 50th birthday, events such as the birth of two daughters and the adoption of a third daughter were reported, and were considered as mildly stressful on the Holmes and Rahe scale. No new somatic event occurred during this life period, and the patient did not consult a physician for mastocytosis. At the age of 51 years, she moved again within Africa for a job (39 LCU) and suffered from her father’s death (63 LCU) (annual stress score = 102 LCU). Subsequently, she developed brown cutaneous lesions related to mastocytosis, treated with corticoids. At the age of 53 years, while the patient and her husband were working in the same company, she realized that he was having an affair with a woman colleague (65 LCU). Then, cutaneous lesions were extended progressively and the patient developed mastocytosis symptoms again. From the age of 55 to 64 years, she suffered chronically from brown maculopapules (red lines), which could have been influenced by family-related events, such as one daughter’s marriage (39 LCU) and a patient’s divorce (65 LCU) (annual stress score = 104 LCU at 56 years old); the death of her mother (63 LCU), the departure of the last daughter (29 LCU), and retirement (45 LCU) (annual stress score = 74 LCU at 62 years old); and the death of the brother (63 LCU)

Analysis of life history and cutaneous symptoms indicated that stressful events could have exacerbated the symptoms of cutaneous mastocytosis (Fig. 1 for details). We observed that, if annual stress score of life events exceeded 50 LCU on the Holmes and Rahe scale, skin manifestations in relation to mastocytosis were induced subsequently. An annual score of 50 LCU could thus be considered as the threshold for stress tolerance of this patient.

**Fig. 2:**
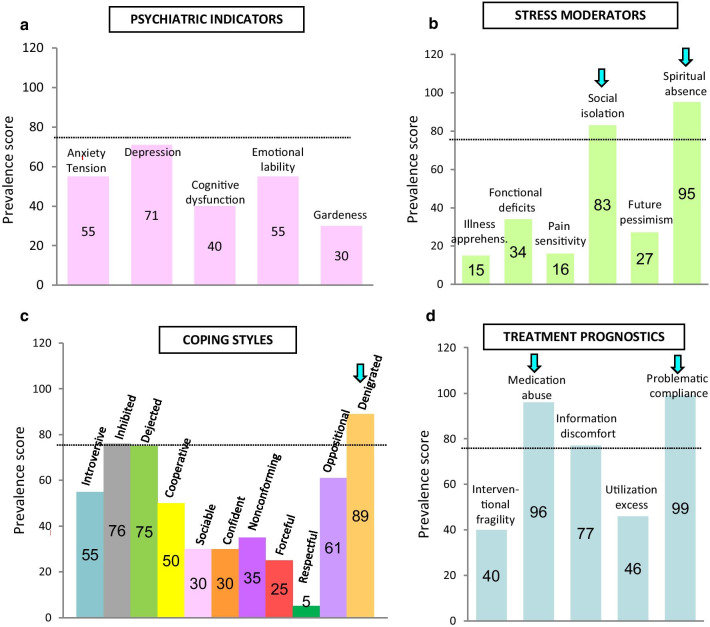
Psychobehavioral diagnosis of the patient. Prevalence scores of psychiatric indicators (**a**), stress moderators (**b**), coping styles (**c**), and treatment prognostics (**d**) are calculated from the Millon Behavioral Medicine Diagnostic questionnaire, which assesses psychobehavioral factors that may affect health. MBMD responses were analyzed and scored according to the Millon guidelines [9]. A prevalence score of up to 75 is considered as significant [10]]. Graphs were built to facilitate visualization of significant factors that may affect health. Turquoise arrows show significant indicators to consider

The MBMD results detailed the psychobehavioral profile of the patient (Fig. 2). This scale identifies attitudes and relationships that may affect health (with significant prevalence score >75) [10]. The psychiatric indicators (anxiety–tension, depression, cognitive dysfunction, emotional lability, guardedness) did not evidence significant psychiatric comorbidities at the time of assessment (Fig. 2a). The MBMD results detailed the stress moderators of the patient (Fig 2b). For the patient, social isolation and the absence of spiritual support reinforced the stressful effect of life events, which could exacerbate the dermatologic symptoms of mastocytosis. Coping styles are shown in Fig 2c. They reflect ways of dealing with life stress and illness. The significant prevalence scores revealed denigrated coping style. This personality trait would predispose the patient to somatic manifestations. In Fig 2d, the significant prevalence score of treatment prognostics indicates the patient’s characteristics that should be considered by the clinicians because they influence treatment outcome. We noticed a low compliance with treatment as well as periods of medication abuse, which did not help care. Regarding everyday behavior, the MBMD test did not reveal negative health habits at that time (alcohol, drugs, eating, caffeine, inactivity, smoking, data not shown.

## Discussion

By synthesizing medical data, life history, psychometric tests, and biological knowledge of the pathology, the points of vulnerability of the patient’s mind–body relationship could be highlighted unambiguously.

Skin manifestations appeared systematically after a period of life events considered as stressful. Personal psychological factors associated with neurobiological processes of stress have surely exacerbated the symptomatology of cutaneous mastocytosis. When events scored up to 50 LCU in the Holmes and Rahe stress scale, the patient systematically displayed brown macropapules on the skin. The coping strategies have certainly been surpassed by stress, as suggested in other pathologies favored by stress susceptibility [13, 14]. The significant stressful life events concerned family bereavements (father and mother) or separations (husband and children). Moreover, the absence of spiritual resources combined with social isolation and noncombative personality traits constitutes a lack of psychological support that could impair stress moderation.

Usually, only physical data are considered in patients suffering from cutaneous pathologies. Neither life history nor stress coping skills are taken into consideration in medical care, while mind–body interactions are essential to health. Considering our results, we can conclude that it is extremely important to take into account the emotional stress and the psychological dimension because they can influence the symptomatology [14–16]. It has been shown, in gut diseases and dermatological pathologies, that stressful events can amplify inflammatory manifestations [15–19]. Moreover, acute stress combined with psychological factors increases the morbidity of allergy and dermatitis by the activation of skin mast cells [17, 20], suggesting psycho-neuro-immunomodulation of cutaneous crises.

**Fig. 3:**
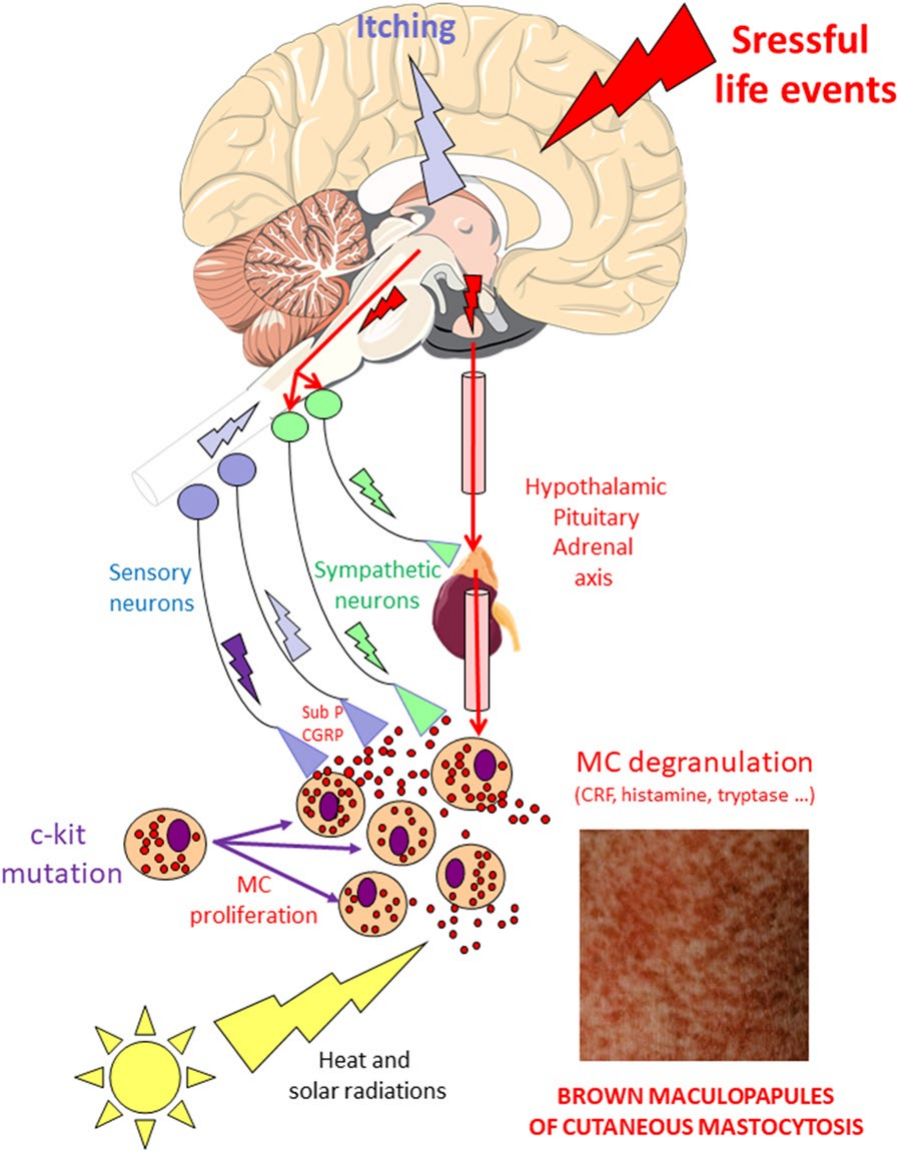
Biological factors and cutaneous symptoms. Cutaneous mastocytosis is characterized by abnormal mast cell (MC) proliferation and degranulation in the skin leading to symptoms such as itchy, brown patches (see photograph). In many cases, mutations in the coding kit gene (c-kit) are responsible for intensification of mast cell proliferation, infiltration, and mediator release in the skin. In the case of this patient, several factors could contribute to the extension of cutaneous symptoms. When the coping strategies of the patient are surpassed, life events induce emotional stress (red flash), implicating the cerebral limbic system and triggering activation of the sympathetic nervous system and the hypothalamic–pituitary–adrenal axis. Heat and sun radiation (yellow flash) can reinforce the activation of mast cells, which locally release several substances such as histamine, tryptase, and corticotropin-releasing factor (CRF), inducing a melanogenic effect. Cutaneous sensory nerve endings, in close contact with mast cells, can also be activated and induce neurogenic inflammation. They mediate the orthodromically itchy reflex associated with prurit (blue flashes). The following antidromic stimulation (violet flash) induces the release of mediators from sensory endings (substance P, calcitonin gene-related peptide CGRP). These neuropeptides cause further mast cell degranulation. All these processes extend and exacerbate cutaneous mastocytosis symptoms

The implication of mind in dermatological symptoms of mastocytosis could be explained by the known biological processes associated with stress (Fig. 3). In the skin, there are anatomical and functional interactions between neurons, blood vessels, and mast cells [2, 21] that could be biological indicators of the influence of mind on the body. Cutaneous mast cells appear to have important physiological functions as sensors of stressful events mediated by the neuroendocrine system [22, 23].

In the case of stressful life events, the brain limbic system involved in emotional and memory processes is activated. This leads to stimulation of the hypothalamic–pituitary–adrenocortical axis and the sympathetic–adrenal–medullary axis. Stress induces the release of neurotransmitters and hormones into the systemic circulation, which alters immune function and skin inflammation [19]. Mast cells occupy a strategic position in the brain–skin axis and release mediators that act in both a paracrine and an autocrine fashion.

Corticotropin-releasing factor (CRF) is considered as a direct mediator of the stress response able to stimulate mast cell degranulation [24, 25]. Mast cells can be activated by acute stress or by intradermal administration of CRF [23, 26]. They strongly express the CRF receptor [27]. The activation of the CRFR-1 receptor on mast cells can lead to strong proinflammatory states and be linked with many cutaneous disorders. Increased vascular permeability and flushing are dependent on CRF receptor expression [28, 29]. CRF produced locally could have melanogenic effects under specific conditions [30]. Solar radiation, for example, can stimulate this CRF production and increase mast cell activation [31, 32]. In the case of this patient, repeated stays in Africa, in addition to stressful life conditions, could have favored the onset of mastocytosis. They would also account for the brown color of maculopapules. This visible component of skin manifestations greatly impacts the human relationships of the patient.

Cutaneous sensory nerve endings located in close contact with mast cells also contribute to the amplification of the symptoms. They are activated by histamine release and mediate the itchy reflex associated with prurit [33]. Moreover they can release neuropeptides in response to stressors. Substance P is a key mediator in connecting the brain and the skin, by stimulating mast cell degranulation during stress, an important process in neurogenic inflammation [26, 33].

The c-kit mutations, solar radiation, stressful events, sympathetic system, HPA axis, circulating hormones, sensory nerves, neuropeptides, and mast cells mediators all could have contributed to the onset of cutaneous symptoms of mastocytosis. They contributed together to the amplification of cutaneous lesions that extended progressively along the life of the patient whenever a stressful event occurred. The lack of spiritual and social coping resources impaired the restoration of healthy mind–body interactions.

Considering the global examination of the case, a positive therapeutic strategy improving mind–body relationship could be proposed to the patient: (1) a therapy reinforcing social and spiritual abilities,to strengthen the capacities of the patient to moderate stress, such as a mindfulness-based intervention program, fostering the patient's ability to introspect and improve psychobehavioral skills and quality of life; (2) careful monitoring of compliance with the prescription of oral H1 antihistamine medication (levocetirizine); and (3) strong discouragement of sun exposure.

This therapeutic strategy could improve harmonious relationships between mind and body, which are essential to staying healthy. The skin is an organ particularly sensitive to imbalances such as those caused by stressful situations.

This article focuses on the psychological aspect of the patient’s experience and its potential role in the exacerbation of cutaneous mastocytosis. Practical and therapeutic implications are explored. However, an anamnestic approach in a single case is not sufficient to demonstrate its validity. This could be a starting point for large-scale studies. This type of neuropsychobehavioral approach should be evaluated on a larger scale in a statistically representative cohort of patients suffering from cutaneous mastocytosis.

## Conclusion

Mastocytosis is a cutaneous pathology that can be considered with regard to the mind–body relationship. Our study underlines the influence of the psyche on mast cell degranulation (psycho-neuro-immunomodulatory pathway) and/or the influence of the skin on the psyche (somatopsychic pathway), which could be implicated in this disease. The transdisciplinary approach presented in this study evaluated the influence of patient-specific factors on cutaneous symptoms. In addition to clinical data, integrative diagnosis has taken into account the patient’s history of life, psychobehavioral skills, and neurobiological processes.

A comprehensive and personalized approach of the patient reveals the vulnerabilities of the connection between the mind and the body and their involvement in the development of disease such as mastocytosis. This type of personalized and integrative diagnosis could be indicated in the case of other chronic diseases resistant to conventional treatments. It could also be proposed in primary health care, especially as a preventive measure throughout life.

## Data Availability

Please contact corresponding author for data requests.
